# Long pentraxin 3 (PTX3) regulates IL-17A-mediated secondary immunity to *Leishmania major* infection in mice

**DOI:** 10.3389/fimmu.2026.1740323

**Published:** 2026-02-10

**Authors:** Gaurav Gupta, Zhirong Mou, Ping Jia, Chukwunonso Onyilagha, Lianyu Shan, Abdelilah Soussi-Gounni, Jude E. Uzonna

**Affiliations:** 1 Department of Immunology, Max Rady College of Medicine, University of Manitoba, Winnipeg, MB, Canada; 2 Department of Medical Microbiology & Infectious Diseases, Max Rady College of Medicine, University of Manitoba, Winnipeg, MB, Canada; 3 Department of Pathology, Max Rady College of Medicine, University of Manitoba, Winnipeg, MB, Canada

**Keywords:** IL-17A, *Leishmania*, memory cells, PTX3, secondary immunity

## Abstract

Cutaneous leishmaniasis, caused by protozoan parasites of the *Leishmania* genus, remains a significant health concern in endemic regions such as the Middle-East, Asia, Latin America, and North Africa. The disease affects millions of people worldwide, with over one million new infections reported annually. Despite its health impact, there is currently no approved vaccine largely due to limited understanding of immunological mechanisms underlying protective immunity and disease pathogenesis. We previously reported that long pentraxin 3 (PTX3), a pattern recognition molecule involved in inflammation, tissue repair, and wound healing, is a negative regulator of immunity in primary *Leishmania major* infection. Specifically, we showed that PTX3 exacerbates disease by suppressing protective Th17 responses. Here, we extend these findings by showing that PTX3 also influences secondary (memory) immunity to *L. major*. *PTX3*-deficient (*PTX3*^-/-^) mice which had resolved a primary infection exhibited enhanced resistance to secondary challenge compared to their wild-type (WT) controls. This enhanced resistance correlated with higher frequencies of effector memory CD4^+^ T cells in the spleens and draining lymph nodes. Upon re-infection, healed *PTX3^-/-^* mice produced significantly more IL-17A, while levels of IFN-γ, TNF-α, and IL-10 were similar. *In vivo* BrdU incorporation assays further revealed increased proliferation of IL-17^+^ CD4^+^ T cells in *PTX3^-/-^* mice. Importantly, neutralization of IL-17A during secondary challenge abolished the enhanced resistance observed in *PTX3^-/-^* mice, confirming a central role of IL-17 in PTX3-regulated secondary immunity. Collectively, our findings identify PTX3 as a key regulator of secondary immunity in cutaneous leishmaniasis and underscores the importance of IL-17 in this process.

## 1 Introduction

Cutaneous leishmaniasis (CL), the most common form of leishmaniasis affecting about a million new individuals annually, causes significant morbidity and mortality, leaving patients with skin ulcers, permanent scars, disability, and social stigma (1–3). Humans and mice that recover from *Leishmania major* infection, one of the species that cause CL, retain a low number of parasites at the primary infection site, promoting durable immunity through IFN-γ-producing CD4^+^ T cells (4). This infection-induced immunity is considered the gold standard for effective vaccines or immunotherapies against CL (5). Therefore, understanding the mechanisms governing its generation, maintenance, and loss is critical for developing successful vaccination strategies.

PTX3 is an acute phase inflammatory protein, comprising of a signal peptide, an N-terminal domain unrelated to any known protein, and a C-terminal pentraxin domain homologous to the short pentraxins, CRP and SAP (6, 7). Expressed by immune and non-immune cells (8), it has a dual role in infection. While protective against bacterial, viral, and fungal pathogens, PTX3 has also been implicated in disease susceptibility (9, 10).

We recently showed that PTX3 is induced during *L. major* infection and suppresses Th17 responses without affecting Th1 immunity (11). Thus, *PTX3*-deficient (*PTX3^-/-^*) mice show enhanced resistance to primary *L. major* infection compared to wild-type controls (11). Although this suppression hinders primary immunity, its role in regulating secondaryimmunity remains unclear. Furthermore, if this enhanced primary immunity does nottranslate to robust secondary immunity, targeting PTX3 may not be a viable therapeutic approach for individuals in endemic regions who face repeated exposures to *Leishmania*.

In this study, we investigated the impact of PTX3 on secondary immunity using *PTX3^-/-^* mice. Our findings demonstrate that PTX3 suppresses optimal secondary immunity to *L. major* re-infection by negatively regulating IL-17A responses, thereby providing new insights into its role in immunity and pathogenesis of cutaneous leishmaniasis.

## 2 Materials and methods

### 2.1 Animals

Heterozygous female *PTX3^+/-^* and homozygous male *PTX3^-/-^* (originally on129SvEv/Bl/6 background and backcrossed for over 10 generations unto C57BL/6 background) mice were bred at the University of Manitoba Central Animal Care Services (CACS) breeding facility. Female homozygous PTX3 deficient (*PTX3^-/-^*) and their age-matched female littermates control mice (6–8 weeks old) were used in the studies. All animal studies were approved by the University of Manitoba Animal Use and Care Committee in accordance with the Canadian Council for Animal Care guidelines.

### 2.2 Parasites

*Leishmania major* parasites (MHOM/IL/80/Friedlin) were grown in M199 medium (Gibco, catalog #11150059) supplemented with 20% heat inactivated fetal bovine serum (FBS) (Hyclone catalog #SH30071.03IH30-45), 2 mM L glutamine (Gibco catalog #25030081), 100 U/ml penicillin, 100 µg/ml streptomycin (Gibco catalog #15140122), 25 mM HEPES (Gibco catalog #15630080) (complete parasite medium). Seven-day stationary phase promastigotes were used for all infection.

### 2.3 Primary infection, secondary challenge, and measurement of DTH response

Groups of WT and *PTX3^-/-^* mice (4–6 mice per group) were infected in the footpads with 1×10^6^ parasites suspended in 50 µl of sterile PBS. For secondary challenge, infected mice were challenged with 1×10^6^ parasites in the contralateral footpads between 12–16 weeks following primary infection when lesions were fully resolved. Lesion development and delayed type hypersensitivity response (DTH) were determined by measuring the thickness of infected footpads with Vernier caliper.

### 2.4 Determination of parasite burden

At 3 weeks after primary or secondary infection, mice were sacrificed and parasite burden in the footpads was determined by limiting dilution as previously described (12). Briefly, the footpads were collected and homogenized in 2 ml complete parasite medium using 15 ml tissue grinders. The suspension was then plated in 96-well plates in triplicates at 10-fold serial dilution, incubated for 7 days at 27°C and assessed for parasite growth under a microscope.

### 2.5 Preparation of soluble *Leishmania* antigen

Soluble *Leishmania* antigen (SLA) used for *in vitro* restimulation of cell cultures was prepared as previously described (13). Briefly, 10^9^ *Leishmania major* stationary-phase L. major promastigotes (MHOM/IL/80/Friedlin) were pelleted by centrifugation and resuspended in Tris-EDTA buffer (100 mM Tris, 1 mM EDTA, pH 8) supplemented with protease inhibitors. Parasites were lysed by sonication, and cellular debris was removed by ultracentrifugation at 15,000 rpm for 30 minutes. The resulting supernatant was clarified by ultracentrifugation at 35,000 rpm for 2–4 hours, followed by overnight dialysis against PBS. The dialyzed preparation was centrifuged at 10,000 rpm for 10 minutes and filtered using a 0.22 um syringe filters to ensure sterility. Protein concertation of the final SLA preparation was determined using the Bradford assay.

### 2.6 *In vitro* recall response and intracellular cytokine staining

At sacrifice, the draining popliteal lymph nodes and spleens from infected WT and *PTX3^-/-^* mice were harvested and made into single-cell suspensions. Cells were washed, resuspended at 5 x 10^6^/ml in complete medium (DMEM (Gibco catalog #12430112 supplemented with 10% heat-inactivated FBS (Hyclone catalog #SH30071.03IH30-45), 2 mM glutamine (Gibco catalog #25030081), 100 U/ml penicillin, and 100 mg/ml streptomycin (Gibco catalog #15140122), and plated at 1 ml/well in 24-well tissue culture plates. Cells were stimulated with SLA, 50 mg/ml for 72 h, and the supernatant fluids were collected and stored at -20°C until assayed for cytokines by ELISA.

For intracellular cytokine analysis, spleen cells and dLNs were assessed for IL-17A, IFN-γ, TNF-α and IL-10 secretion directly *ex vivo* by flow cytometry following 4 hr *in vitro* stimulation with Cell activation cocktail with Brefeldin A (Biolegend catalog #423304). Thereafter, the cells were routinely stained for surface marker expression, permeabilized with 0.1% saponin (Sigma-Aldrich catalog #S7900-25G) in staining buffer and then stained with specific fluorochrome-conjugated mAbs against IL-17A (clone: TC11-18H10.1), IFN-γ (clone: W18272D), TNF-α (clone: MP6-XT22) and IL-10 (clone: JES5-16E3) (BioLegend, catalog #506916, 163504, 506308, 505008). Samples were acquired on a BD FACS Cantor machine and analyzed using FlowJo software (TreeStar Inc, Ashland, OR).

### 2.7 Quantification of transcript levels by RT-PCR

Total RNA was extracted from murine foot pad using the Trizol (Sigma). mRNA was reverse transcribed and cDNA was amplified by RT-PCR using SYBR Green chemistry as described previously (14). Murine primers (PTX3 and β-actin) and reaction conditions were obtained from the PRIMER BANK website http://pga.mgh.harvard.edu/primerbank (Massachusetts General Hospital Primer Bank). Data were normalized to the housekeeping gene β-actin and presented as fold induction over naïve uninfected foot pad cells using the delta-delta CT method. Mouse Forward PTX3-CCTGCGATCCTGCTTTGTG (Primer Bank ID: 31982085a1), Mouse Reverse PTX3-GGTGGGATGAAGTCCATTGTC Primer Bank ID: 31982085a1, Mouse Forward β-actin-AGGAAGTCCCGAAGCAAGAGA (Primer Bank ID: 33585589a1, Mouse Reverse β-actin-CTCCAGTGATTTCTGTGGCAA Primer Bank ID: 33585589a1).

### 2.8 Cytokine ELISAs

IL-17A, IFN-γ, TNF-α and IL-10 concentrations in cell culture supernatant fluids were measured by sandwich ELISA using Ab pairs from BD Pharmingen (Mouse IFN-γ ELISA Kit catalog #558258) or Biolegend (ELISA MAX Standard Set mouse IL-17A catalog #432501, ELISA MAX Standard Set mouse IL-17A catalog #431411), according to manufacturer’s suggested protocols. The sensitivities of the ELISAs were15 pg/ml, 7.5 pg/ml, I0 pg/ml, and 7.5 pg/ml for IL-17A, IFN-γ, TNF-α, and IL-10, respectively.

### 2.9 *Ex vivo* assessment of memory T cell and Treg subsets

*L. major* infected WT and PTX3^-/-^ mice were sacrificed at 12 weeks post-infection and single cell suspensions of the draining lymph nodes and spleens were made. The cells were counted and adjusted to 5 × 10^6^/ml and 100 µl aliquots were stained directly *ex vivo* with different flourochrome-conjugated mAbs against CD3 (clone: 145-2C11), CD4 (clone:

GK1.5), CD44 (clone: NIM-R8), CD62L (clone: MEL-14), CCR7 (clone: 4B12) and CD127 (clone:SB/199) (Biolegend, catalog #100334, 100422, 156008, 104412, 120115 and 121111) and analyzed by flow cytometry. In some experiments, t-distributed Stochastic Neighbor Embedding (tSNE) analysis was performed using FlowJo v10 (FlowJo LLC, Ashland, OR) to visualize high-dimensional flow cytometry data. After gating on live single cells, CD3^+^CD4^+^ T cells were identified and exported, and 2,000 events per sample were randomly downsampled to standardize cell numbers across groups. Dimensionality reduction incorporated the surface markers CD44, CD62L, CCR7, and CD127. t-SNE was run using default settings with 1,000 iterations and a perplexity of 30, and the resulting two-dimensional embeddings were used to display population distributions. Analyses and overlays were annotated according to sample ID.

For Treg staining, spleens and dLN cells were directly stained *ex vivo* for surface expression of CD3 (clone: 145-2C11), CD4 (clone: GK1.5) and CD25 (clone:PC61) (Biolegend, catalog #100334, 100406, 102012). Thereafter, they cells were stained for intracellular expression of Foxp3 using Treg staining kit (eBioscience, catalog # 00-5523-00) according to the manufacturer’s suggested protocol.

### 2.10 Ki67 staining and *in vivo* BrdU labeling

Naïve and healed WT and *PTX3^-/-^* mice were injected (ip) with 2 mg BrdU/mouse and challenged with 1 × 10^6^ *L. major* parasites in the contralateral footpad the next day. BrdU injection was repeated on days 4 and 6 post-infection and mice were sacrificed on day 7 post-infection to assess cytokine production (IL-17A, IFN-γ, TNF-α and IL-10) by proliferating (i.e., BrdU^+^ or Ki67^+^) CD4^+^ T cells in the dLNs. BrdU and Ki67 intracellular staining were performed with kits/antibodies from BD Pharmingen (catalog# 559619) and Biolegend (catalog# 652422).

### 2.11 *In vivo* neutralization of IL-17A

Naïve and healed mice (WT and *PTX3^-/-^*) were treated with either control immunoglobulin (isotype Ig) or anti-IL-17A, neutralizing monoclonal antibodies (0.5 mg/mouse, i.p, all purchased from BioXcell, anti-IL-17A (clone 17F3) catalog# BE0173 and anti-IgG1 isotype (clone: MOPC-21)-catalog# BE0083) 1 day before challenge infection with *L. major*. Antibody treatment was continued once weekly at 0.5 mg/mouse for additional 3 weeks. Mice were sacrificed after 3 weeks post infection to determine parasite burden.

### 2.12 Statistics

Results are presented as means ± standard error (SE). Comparisons between groups were performed using paired Student’s *t*-test or one-way and two-way analysis of variance (ANOVA) with Tukey *post hoc* correction for multiple comparisons, as appropriate. A *p* value ≤0.05 was considered statistically significant.

## 3 Results

### 3.1 *PTX3^-/-^* mice display enhanced resistance to secondary *L. major* infection

**Figure 1:**
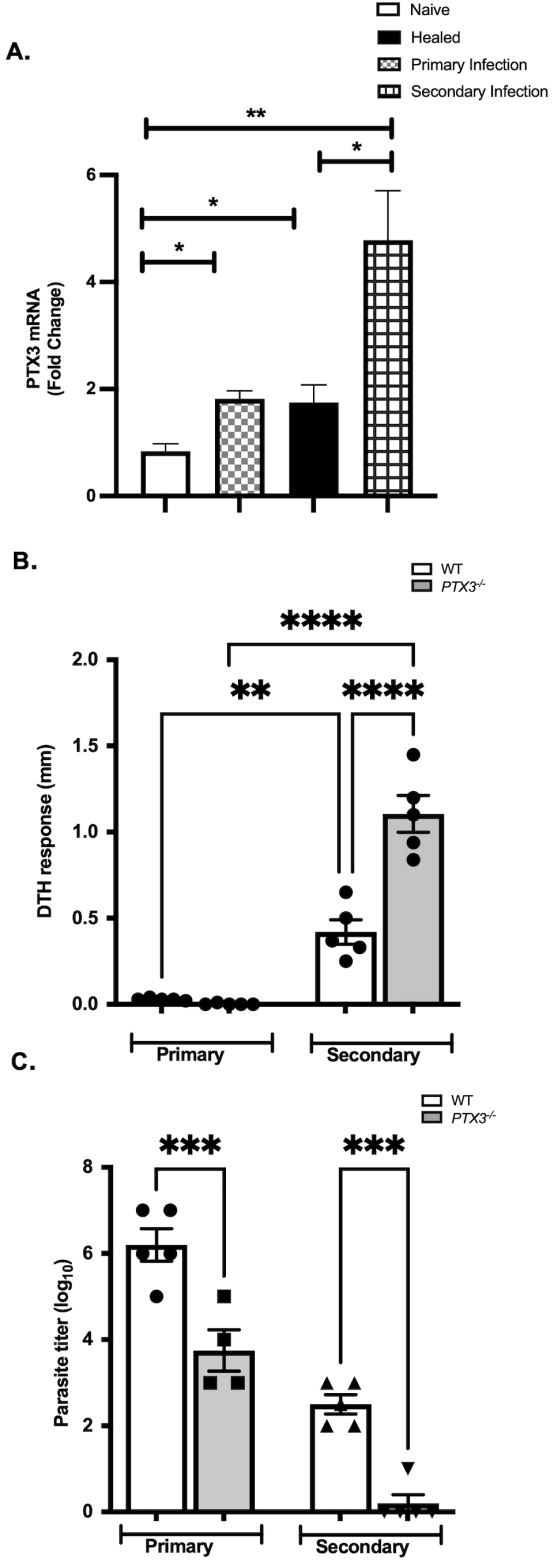
Elevated PTX3 expression at the site of secondary *L. major* challenge is associated with reduced delayed-type hypersensitivity response and increased parasite burden. Naïve or healed (previously infected with *L. major*) C57BL/6 mice (n = 5) were injected in the footpads with PBS or infected with 1 x 10^6^ stationary-phase *L. major* promastigotes (contralateral footpads for healed mice). After 7 days, mice were sacrificed and PTX3 mRNA expression at the infection site was assessed by RT-PCR **(A)**. WT and *PTX3*^-/-^ mice were infected with 1×10^6^ stationary-phase *L. major* promastigotes in the footpads and allowed to heal (> 12 weeks). Healed mice, along with their age-matched naïve controls, were then challenged with *L. major* (contralateral for healed mice). After 72 hours, footpad swelling (a measure of delayed-type hypersensitivity, DTH response) was measured with digital calipers **(B)**. Three weeks after challenge, parasite burden in the infected footpads was quantified by limiting dilution **(C)**. Data represent means ± standard error (n = 5 mice per group) and are representative of 2 independent experiments with similar results. *p < 0.05; **p < 0.01 ***p < 0.005; ****p < 0.0001; ns Not Significant, ND, No parasites detected.

Recent studies by our group have shown that PTX3 negatively regulates primary immunity to *L. major* infection (11). To assess whether PTX3 also modulates secondary (memory) immunity *L. major*, we first determined changes in the levels of PTX3 gene expression following secondary *L. major* challenge in previously infected and healed mice. Qualitative RT-PCR analysis showed a significant upregulation of PTX3 mRNA at the site of secondary *L. major* infection (**Figure 1A**), suggesting that, as in primary infection, PTX3 may also influence immunity during secondary immunity.

To determine this, we challenged healed wild-type (WT) and PTX3-deficient (*PTX3^-/-^*) mice with *L. major* and compared their delayed-type hypersensitivity (DTH) responses which is a surrogate marker of memory CD4^+^ T cell-mediated immunity. At 3 days post-secondary challenge, healed *PTX3^-/-^* mice displayed a significantly stronger DTH response compared to healed WT mice (**Figure 1B**). This heightened DTH response in healed *PTX3^-/-^* mice was associated with significantly lower parasite burden at this site of infection compared to WT healed mice at 3 weeks post *L. major* challenge (**Figure 1C**).

**Figure 2:**
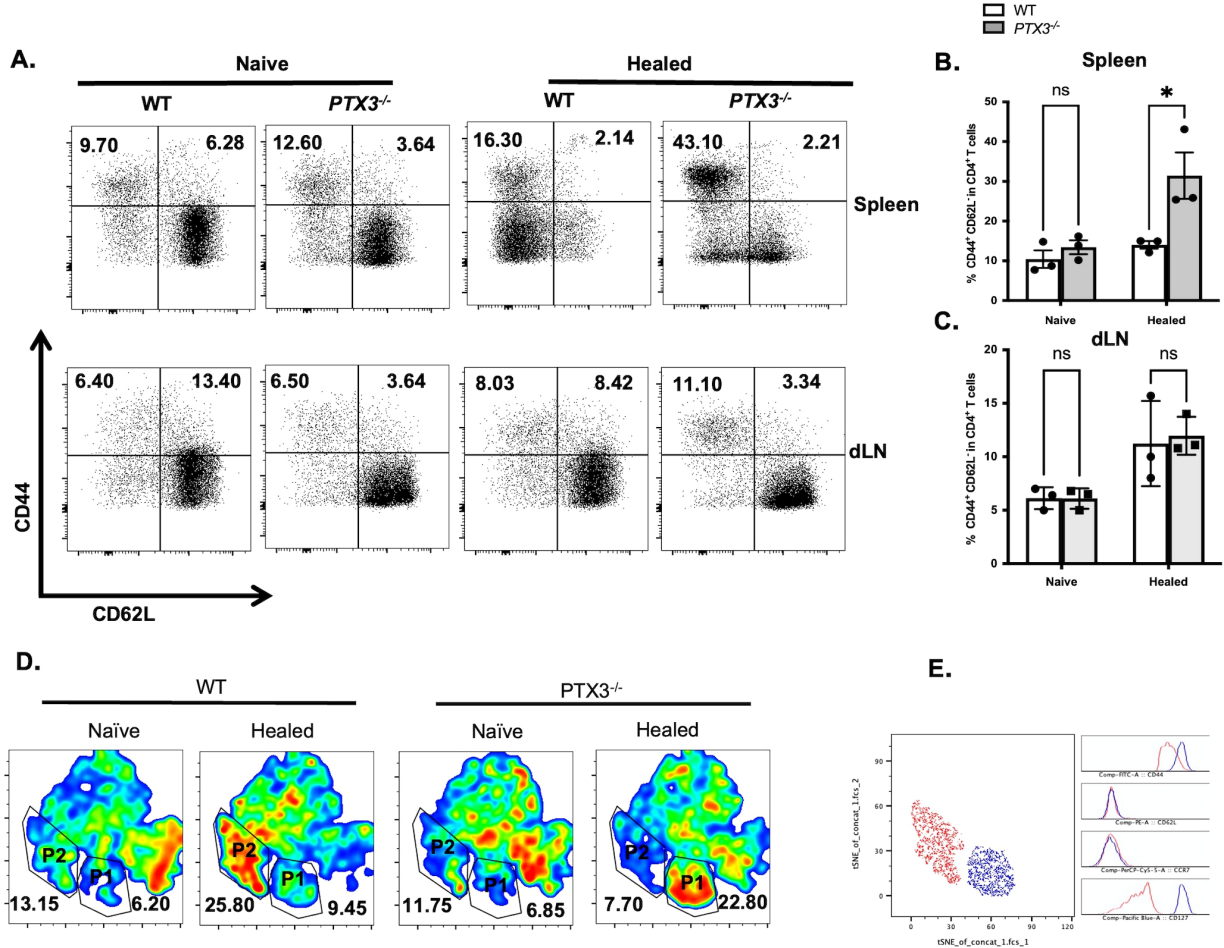
Increased frequency of splenic effector memory-like CD4^+^ T cells in *L. major* infected and healed *PTX3^-/-^* mice. Wild type (WT) and *PTX3^-/-^* were infected with *L. major* and allowed to heal (>12 weeks after infection). Healed and aged matched naïve control mice were sacrificed, and the frequencies of CD44^+^CD62L^-^ (effector memory-like) CD4^+^ T cells were analyzed directly *ex vivo* by flow cytometry in their spleens **(A, B)** and draining lymph nodes (dLN, **A**, **C**) cells by gating on CD3^+^CD4^+^ cells. A tSNE plot of CD4^+^ CD44^+^ T cell clustering from the spleen of healed and aged matched naïve WT and *PTX3^-/-^* mice is shown in **(D)** along with the expression profiles of CD44, CD62L, CCR7 and CD127 **(E)**. Data are presented as means ± standard error and are representative of 2 independent experiments (n = 3 mice per group) with similar results. *p < 0.05. ns, Not significant.

Given that DTH responses are primarily mediated by antigen-experience CD4^+^ T cells, which are critical for infection-induced resistance in cutaneous leishmaniasis (15), we hypothesized that healed *PTX3^-/-^* mice would have higher frequency of different subsets of memory CD4^+^ T cells in their spleens and draining lymph nodes compared to their WT counterpart mice. Flow cytometric analysis (**Figure 2**; **Supplementary Figure S5**) showed that healed *PTX3^-/-^* mice had significantly higher frequency of splenic effector memory-like (CD44^+^CD62L^-^) CD4^+^ T cells in their spleens compared to their WT controls. In contrast, the percentage of central memory-like (CD44^+^CD62L^+^) CD4^+^ T cells were comparable between the two groups (**Figures 2A–C**). To further characterize the distribution of memory CD4^+^ T cell populations in the spleen, we employed t-distributed stochastic neighbor embedding (tSNE) analysis, which clearly identified two distinct clusters designated P1and P2. The P1 cluster was more prominent in healed *PTX3^-/-^* mice, whereas the P2 cluster was enriched in healed WT mice (**Figure 2D**). Phenotypic profiling showed that P1 cells expressed higher levels of CD44 and CD127, markers associated with effector memory and long-term survival. In contrast, CD62L and CCR7 expression levels were comparable in both P1 and P2 clusters (**Figure 2E**). Collectively, these findings indicate that PTX3 regulates immunity to secondary *L. major* infection, consistent with its previously described role in regulating immunity during primary infection.

### 3.2 Enhanced secondary immunity in PTX3 deficient mice is not associated with increased IFN-γ production

**Figure 3:**
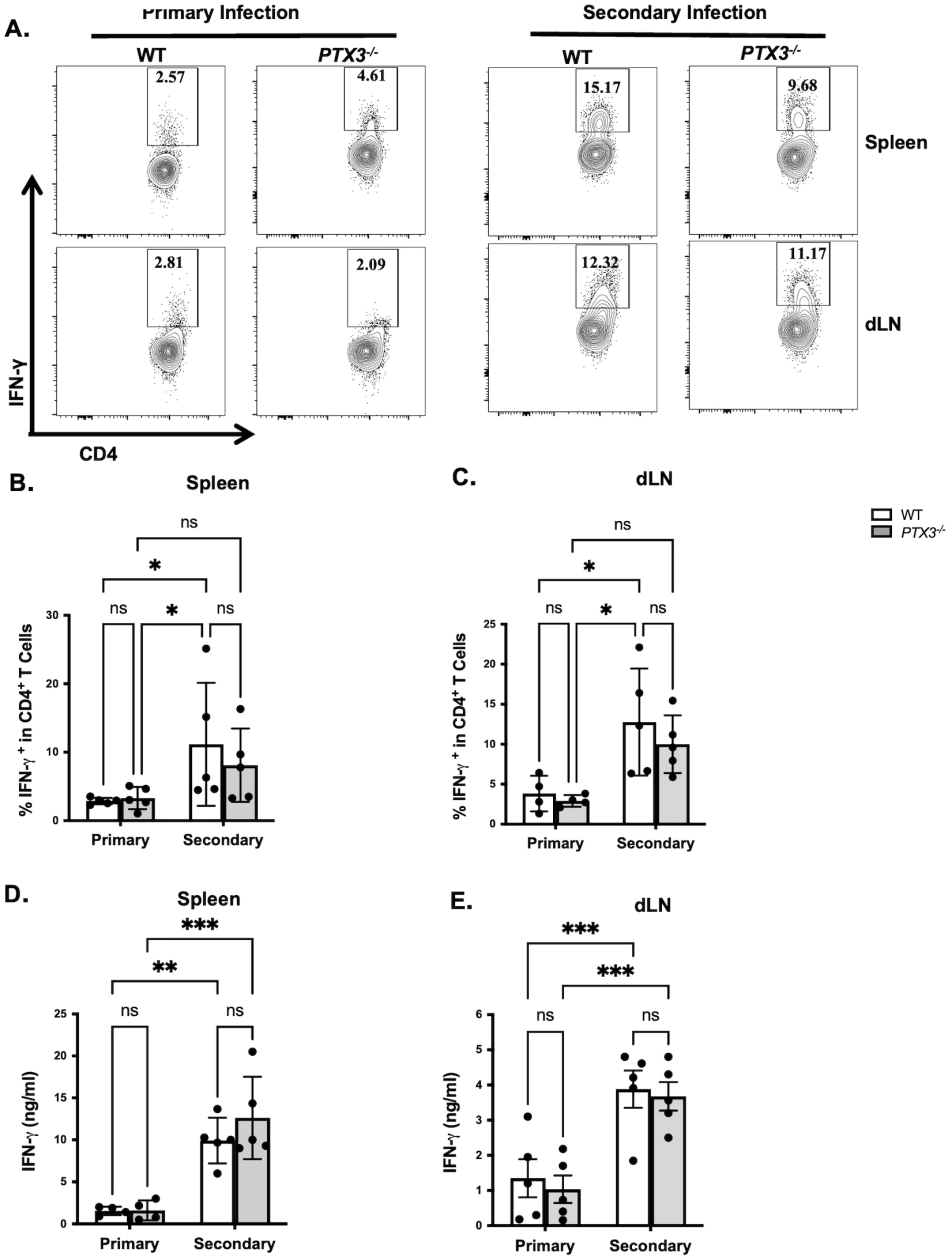
PTX3 deficiency does not affect IFN-γ response during secondary *L. major* infection. Healed and age-matched naïve wild-type (WT) and *PTX3*^-/-^ mice were infected in the footpad (contralateral for healed mice) with 1x10^6^ stationary-phase *L. major* promastigotes. Three weeks post-infection, mice were sacrificed and the frequency of IFN-γ-producing CD4^+^ T cells in the spleen **(A, B)** and dLN **(A, C)** were analyzed directly *ex vivo* by flow cytometry by gating on CD3^+^CD4^+^ T cells. Additionally, single cell suspensions from spleen **(D)** and dLN **(E)** cells were restimulated *in vitro* with soluble leishmania antigen (SLA, 50 μg/ml) for 72 hr and IFN-γ levels in the culture supernatant fluids were measured by ELISA. Data are presented as means ± standard error and are representative of 2 independent experiments (n = 5 mice per group) with similar results. *p < 0.05; **p < 0.01 ***p < 0.005; ns, Not significant.

Secondary immunity to *L. major*, (also known as infection-induced resistance) is primarily mediated by IFN-γ produced by CD4^+^ T cells (4, 16, 17). Consistent with this, we observed both healed WT and *PTX3^-/-^* mice upon secondary *L. major* challenge exhibited increased frequencies of IFN-γ^+^CD4^+^ T cells in their spleen and draining lymph nodes (dLNs) and their cultures produced significantly higher levels of this cytokine compared to their primary-infected control mice (**Figures 3A–E**; **Supplementary Figure S6**). However, there were no significant differences in the frequency of IFN-γ^+^CD4^+^ T cells and levels of IFN-γ in spleen and dLN culture supernatant fluids of WT and *PTX3^-/-^* mice following secondary *L. major* challenge (**Figures 3A–E**; **Supplementary Figure S6**). These findings suggest that the enhanced resistance observed in healed *PTX3^-/-^* mice following secondary challenge was not due to enhanced Th1 response, implicating alternative immune mechanisms in PTX3-regulated secondary protection.

### 3.3 Enhanced secondary immunity in PTX3 deficient mice is not associated with increased TNF-α and IL-10 production

Both TNF-α (18) and IL-10 (4, 19) have also been implicated in regulating secondary immunity in CL. To assess whether differential production of these cytokines contribute to the enhanced protection observed in healed *PTX3^-/-^* mice, we compared their expression in healed WT and *PTX3^-/-^* mice following secondary *L. major* challenge. Flow cytometric analysis revealed no significant differences in the frequencies of TNF-α^+^CD4^+^ T cells (**Supplementary Figures S1A–C**, **S6**) and IL-10^+^CD4^+^ T cells (**Supplementary Figures S2A–C**, **S6**) between the two groups. Similarly, levels of TNF-α and IL-10 in the supernatants of spleen and draining lymph node cell cultures were comparable in WT and *PTX3^-/-^* mice (**Supplementary Figures S1D, E**, **2D, E**, respectively).

Given the established role of IL-10-producing Foxp3^+^CD4^+^ regulatory T cells (Tregs) in suppressing secondary immunity to *Leishmania major* (19–21), we also assessed Treg frequencies. However, the frequency of Tregs in the spleens (**Supplementary Figures S3A, B**, **S6**) and dLN (**Supplementary Figures S3A–C**, **S6**) were similar between WT and *PTX3^-/-^* mice following secondary *L. major* challenge. Taken together, these findings suggest that the enhanced resistance observed in healed *PTX3^-/-^* mice following secondary *L. major* challenge is unlikely to be due to altered production of TNF-α or IL-10 in healed, or due to differences in the frequency of regulatory T cells. This supports the notion that other immune pathways, such as IL-17A responses, may underlie the improved secondary immunity in the absence of PTX3.

### 3.4 Enhanced secondary immunity in PTX3 deficient mice is associated with increased IL-17A production

**Figure 4:**
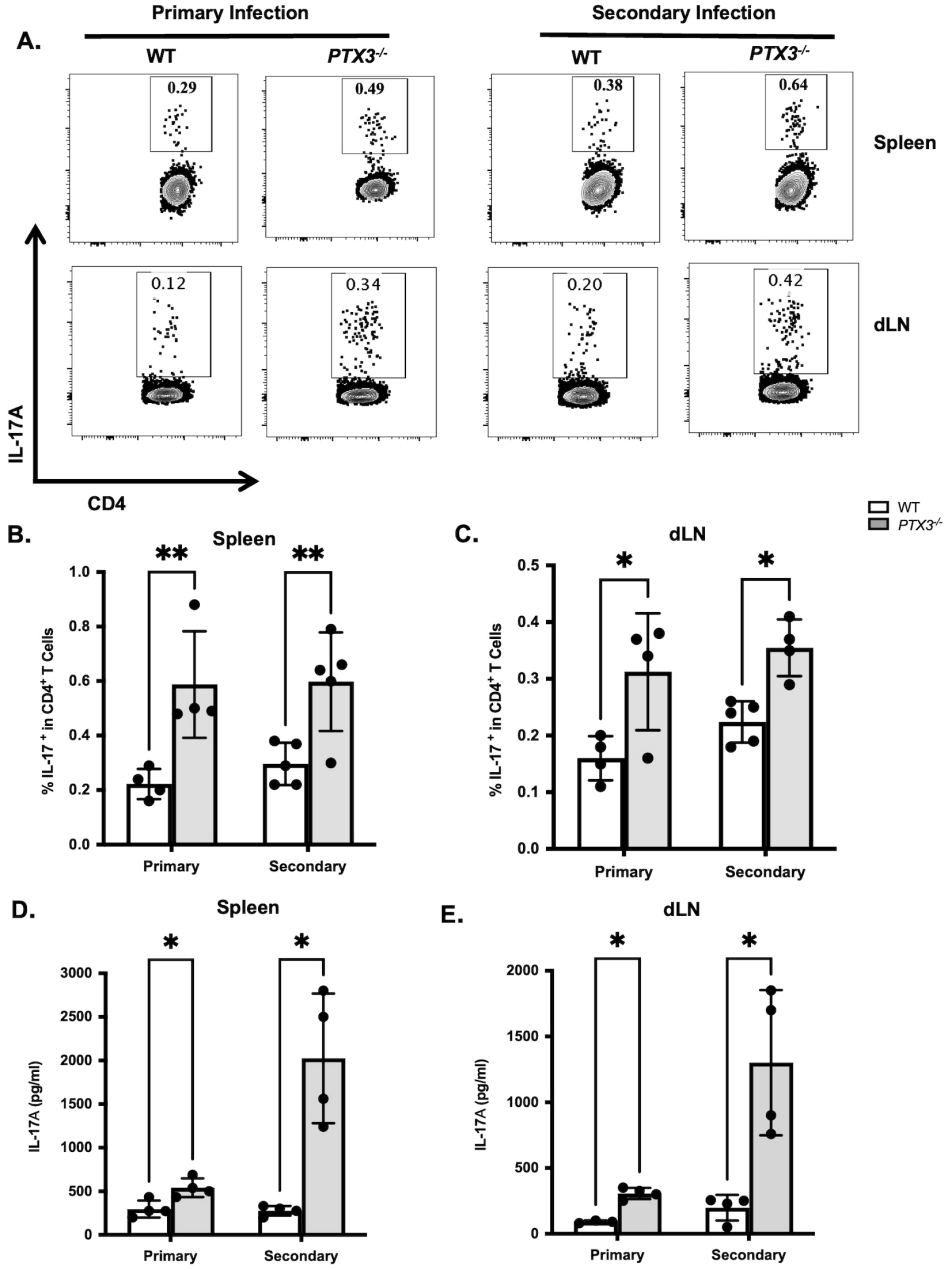
PTX3 deficiency enhances Th17 and IL-17 responses during secondary *L. major* infection. Healed and age-matched naïve wild-type (WT) and *PTX3*^-/-^ mice were infected in the footpad (contralateral for healed mice) with 1x10^6^ stationary-phase *L. major* promastigotes. Three weeks post-infection mice were sacrificed and the frequency of IL-17^+^ -producing CD4^+^ T cells in the spleen **(A, B)** and dLN **(A, C)** were analyzed directly *ex vivo* by flow cytometry by gating on CD3^+^CD4^+^ T cells. Single cell suspension from spleen **(D)** and dLN **(E)** were also restimulated *in vitro* with SLA (50 μg/ml) for 72 hr and the levels of IL-17 in the culture supernatant fluids were determined by ELISA. Data are presented as means ± standard error and are representative of 2 independent experiments (n = 5 mice per group) with similar results. *p < 0.05; **p < 0.01. ns, Not significant.

We previously reported that PTX3 impairs optimal primary immunity to *L. major* by suppressing IL-17A production by CD4^+^ T cells (11). Based on these findings, we hypothesized that the enhanced secondary immunity observed in *PTX3^-/-^* mice would be similarly associated with increased IL-17A production. Consistent with this hypothesis, flow cytometric analysis revealed that healed *PTX3^-/-^* mice had significantly higher frequency of IL-17A^+^ CD4^+^ T cells in both the spleen and dLN (**Figures 4A–C**; **Supplementary Figure S6**) compared to secondary challenged WT mice. In addition, culture supernatants from spleen (**Figure 4D**) and dLN (**Figure 4E**) cells showed markedly elevated levels of IL-17A in *PTX^-/-^* mice relative to controls WT controls. Interestingly, IL-17A levels were also higher in spleen and dLN cell culture supernatants of secondary-infected healed *PTX3^-/-^* mice compared to primary infected *PTX3^-/-^* mice. However, this difference was not observed in direct *ex vivo* flow cytometric analysis, likely due to stimulation with soluble *leishmania* antigen during *in vitro* cultures which could amplify antigen-specific IL-17A response.

### 3.5 Higher frequency of CD4^+^ T cells in healed *PTX3^-/-^* following secondary *L. major* challenge

**Figure 5:**
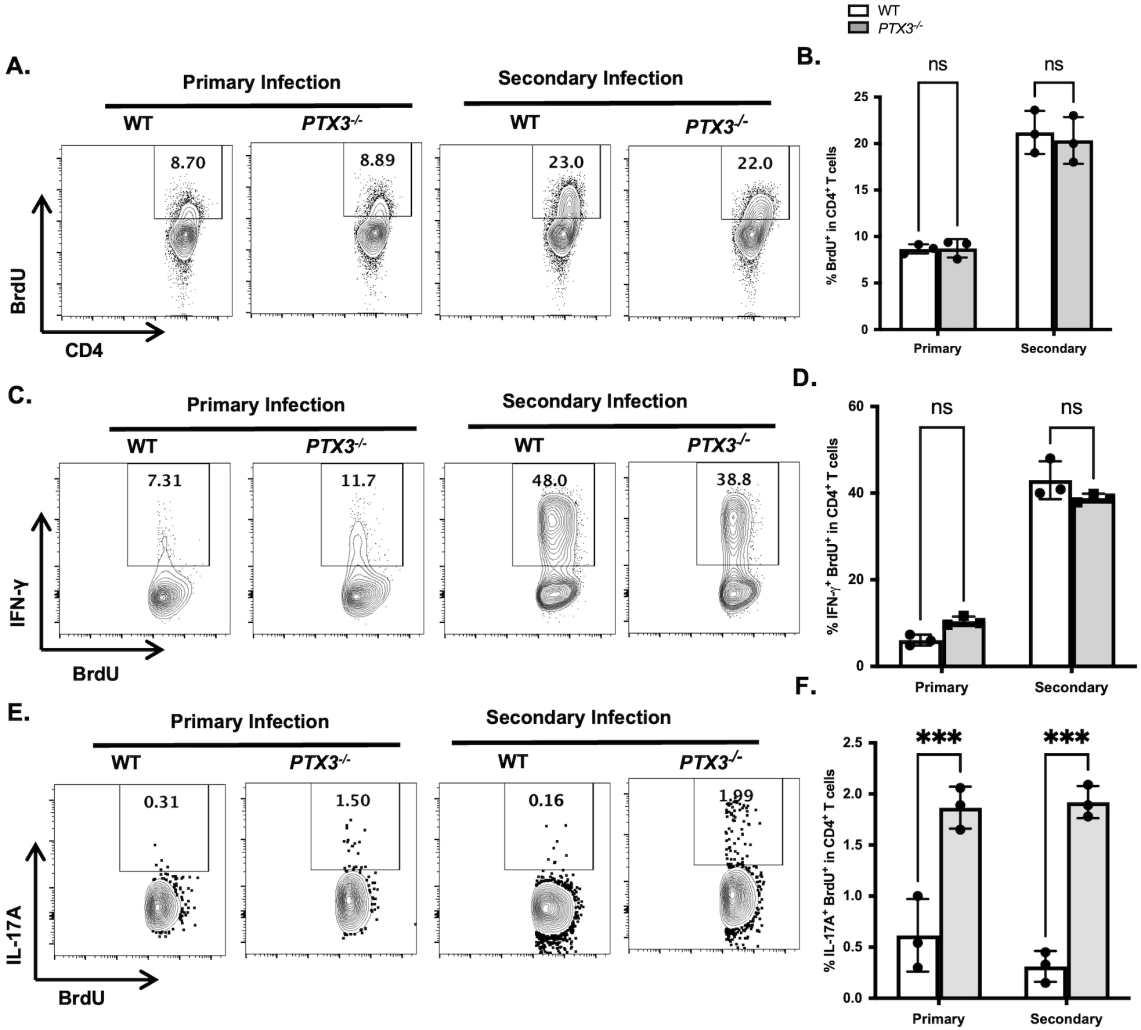
PTX3 deficiency enhances *in vivo* recall of antigen experienced IL-17^+^CD4^+^ T cells during secondary *L. major* infection. WT and *PTX3*^-/-^ were infected in the footpad with 1×10^6^ stationary-phase *L. major* promastigotes and allowed to heal (> 12 weeks). Healed mice and their aged matched naïve control mice were injected intraperitoneally with BrdU (2 mg/100 μl) one day prior to challenge with *L. major* (contralateral footpad for healed mice). A second BrdU injection was administered on Day 4 post challenge, and mice were sacrificed 3 days later. The frequency of proliferating (BrdU^+^) CD4^+^ cells in the dLN **(A, B)** was analyzed by *ex vivo* by flow cytometry. The frequency of proliferating and cytokine producing cells (IFN-γ^+^BrdU^+^ and IL-17A^+^BrdU^+^) in the dLN was also analyzed **(C-F)**. Data are presented as means ± standard error and are representative of 2 independent experiments (n = 3 mice per group) with similar results. ***p < 0.005; ns, Not significant.

Next, we assessed and compared the *in vivo* proliferative and cytokine producing capacities of CD4^+^ T cells from healed WT and *PTX3^-/-^* mice following *L. major* challenge. Analysis of dLN showed that both healed WT and *PTX3^-/-^* mice showed increased but comparable frequency of BrdU^+^CD4^+^ T cells (**Figures 5A, B**; **Supplementary Figure S7A**) and IFN-γ^+^BrdU^+^CD4^+^ T cells (**Figures 5C, D**; **Supplementary Figure S7A**), relative to their primary infection controls. In contrast and consistent with direct *ex vivo* and *in vitro* antigen stimulation, healed *PTX3*^-/-^ mice showed significantly higher frequency of IL-17^+^BrdU^+^CD4^+^ T cells in the dLN compared to their WT healed mice (**Figures 5E, F**; **Supplementary Figure S7A**). This was further supported by Ki67 staining, which showed an increased frequency of IL-17^+^Ki67^+^CD4^+^ T cells in the dLN of *PTX3^-/-^* mice compared to their WT controls (**Supplementary Figure S4**, **S7B**). Together, these findings show that antigen-experienced CD4^+^ T cells in healed *PTX3^-/-^* mice proliferate into IL-17A-producing effector cells upon secondary *L. major* challenge, further implicating IL-17A as a key mediator of enhanced secondary (memory) immunity in the absence of PTX3.

### 3.6 Enhanced resistance to secondary *L. major* infection in PTX3^-/-^ mice is due to increased IL-17A response

**Figure 6:**
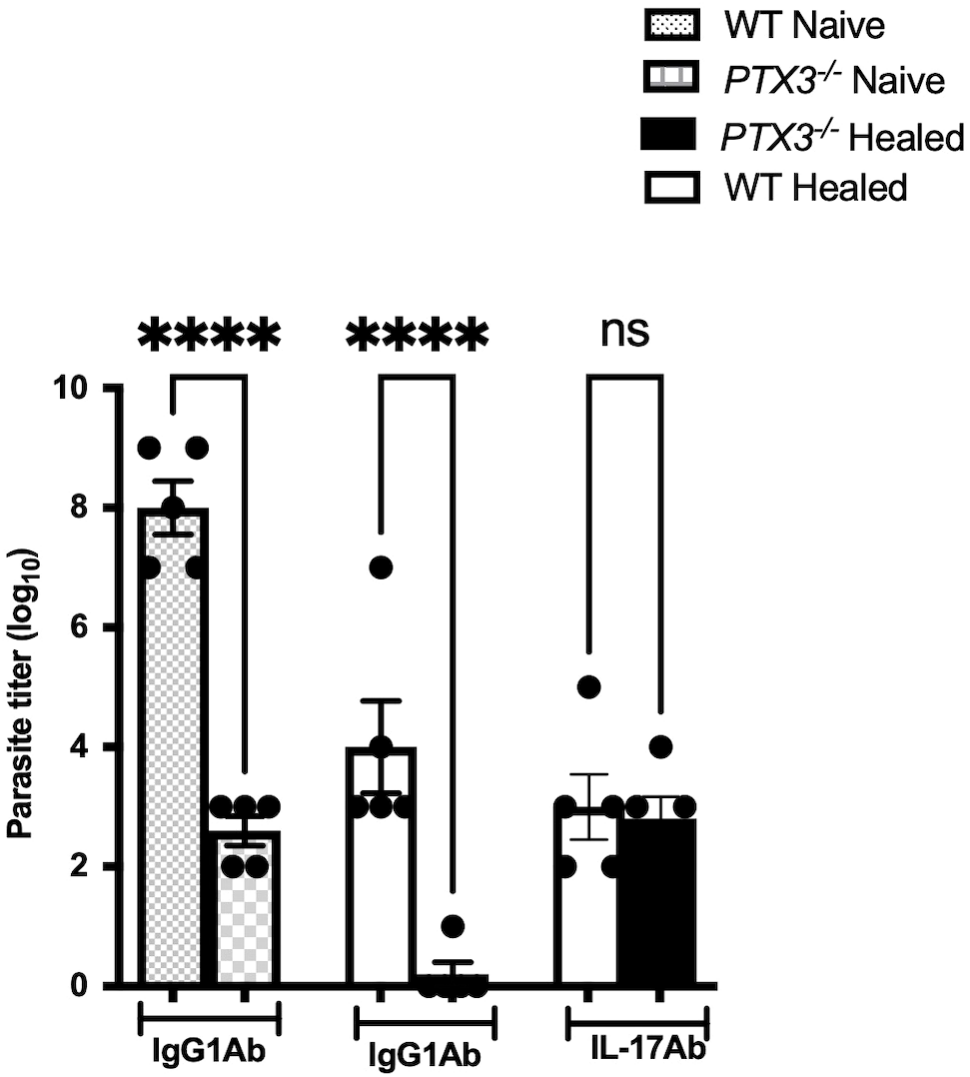
Neutralization of IL-17A abolished enhanced immunity in healed *PTX3^-/-^* mice during secondary *L. major* infection. Healed and age-matched naïve WT and *PTX3*^-/-^ mice were treated intraperitoneally with either an isotype control Ig or anti-IL-17A neutralizing monoclonal antibody (mAb) one day prior to secondary challenge with 1x10^6^ stationary phase *L. major* promastigotes. Anti-IL17 mAb treatment was continued once weekly for additional 3 weeks. Mice were sacrificed 4 weeks post-infection, and parasite burden in the infected footpads was quantified. Data are presented as means ± standard error and are representative of 2 independent experiments (n = 5 mice per group) with similar results. ****p < 0.0001; ns, Not significant.

**Figure 7:**
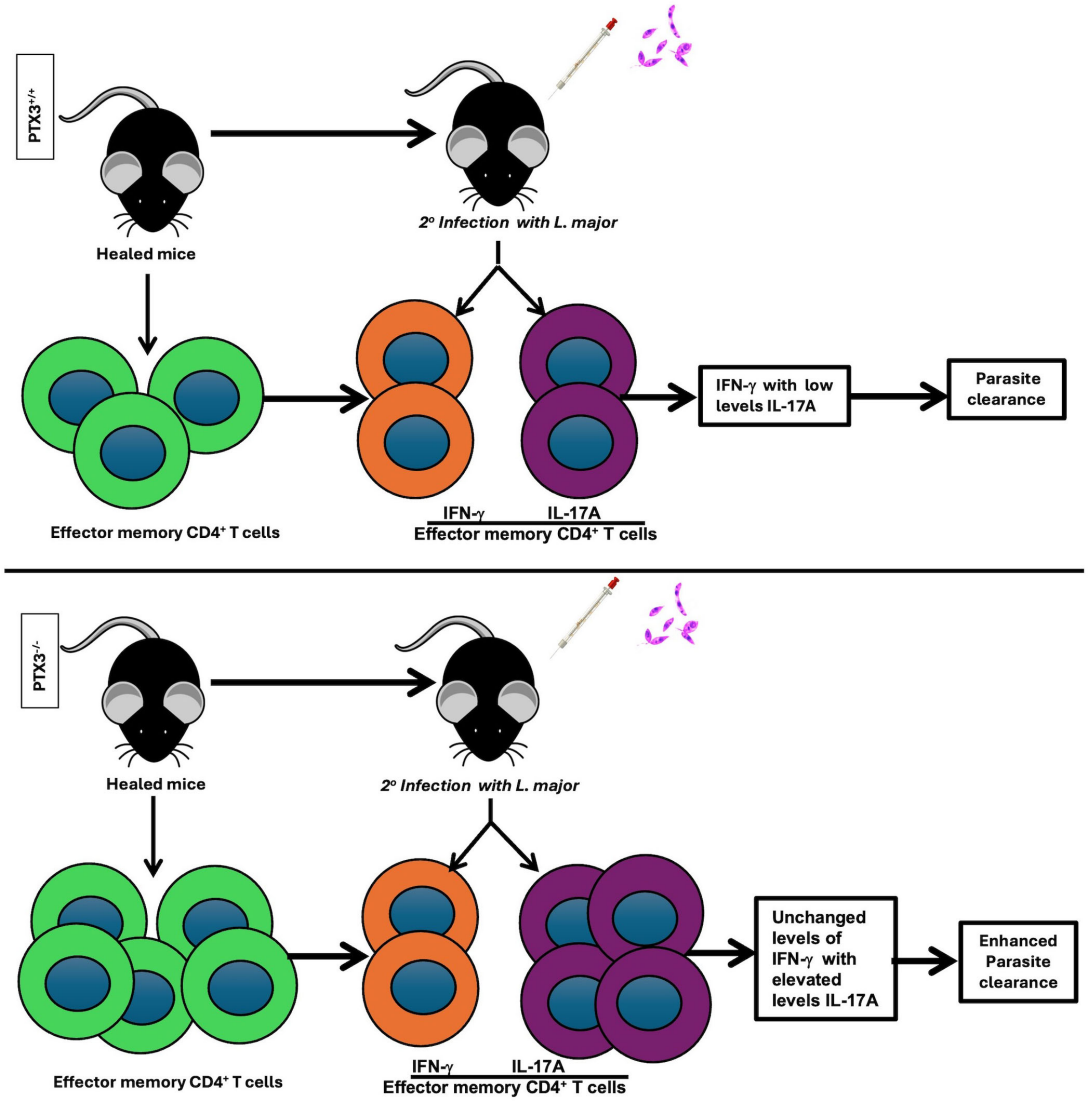
Proposed mechanistic model of PTX3 regulation of secondary immunity to *Leishmania major.* In a PTX3 sufficient mice, healing from *Leishmania major* infection results in relatively low frequency of effector memory CD4^+^ T cells, suggesting that PTX3 may negatively regulate expansion of these subsets of memory cells. Upon secondary *L. major* challenge, these memory cells predominantly differentiate into IFN-γ producing CD4^+^ T cells. However, the presence of PTX3 limits the development of IL-17A-producing effector CD4^+^ T cells resulting in lower IL-17A production and reduced IL-17A-mediated effector activity. In contrast, PTX3 deficient mice exhibit increased frequencies of effector memory-like CD4^+^ T cells following primary infection. Upon secondary *L. major* challenge, these cells proliferate robustly and differentiates into higher frequency of IL-17A-producing CD4^+^ T cells. This elevated IL-17A together with IFN-γ synergistically enhances parasite killing leading to increased host resistance. Thus, in the absence of PTX3, the augmented IL-17A response contributes to improved resistance during secondary infection.

Given that the enhanced resistance of healed *PTX3^-/-^* mice to secondary *L. major* infection was not associated with higher IFN-γ and TNF-α responses compared to WT controls, we postulated that the enhanced resistance was instead mediated by increased IL-17A production. To test this, we performed *in vivo* neutralization of IL-17A in both healed WT and *PTX3^-/-^* mice following secondary *L. major* challenge and assessed parasite burden (**Figure 6**). Neutralization of IL-17A in healed *PTX3^-/-^* mice abrogated their enhanced secondary immunity as evidenced by significantly higher parasite burden compared to isotype-treated controls (**Figure 6**). Collectively, these findings show that the enhanced secondary immunity observed PTX3^-/-^ mice is dependent, at least in part, on increased IL-17A production that likely act in concert with IFN-γ to promote more effective parasite control (**Figure 7**).

## 4 Discussion

Our study identifies PTX3 as a key negative regulator of secondary immunity to *Leishmania major*. We demonstrate that PTX3 expression is elevated during secondary *Leishmania major* infection, and that its absence (by genetic deletion) results in enhanced protective immunity as evidenced by reduced parasite burden, heightened delayed-type hypersensitivity and increased frequencies of effector memory CD4^+^ T cells. Mechanistically, this enhanced protection was associated with increased production of IL-17A, which acts synergistically with IFN-γ to promote more effective parasite control. This is the first study showing the importance of IL-17A in mediating enhanced secondary immunity to *Leishmania* infection (**Figure 7**).

It is well established that IFN-γ-producing CD4^+^ T cells are essential for mediating infection-indued immunity in cutaneous leishmaniasis (16). Depletion of CD4^+^ T cells or neutralization of IFN-γ abolish resistance to infection (22, 23). In the current study, both WT and PTX3-deficient mice mounted strong and comparable IFN-γ response in the spleen and dLN following secondary *L. major* challenge. In addition, there were no significant differences in the frequency of regulatory T cells or IL-10-producing CD4^+^ T cells between healed WT and PTX3^-/-^ mice. These observations suggest that the enhanced resistance observed in healed PTX3^-/-^ mice following secondary *L. major* challenge is not attributable to enhanced production of IFN-γ by CD4^+^ T cells or reduction in the production of deactivating cytokines such as IL-10 or frequency of regulatory T cells, both of which have been shown to modulate protective immune response in cutaneous leishmaniasis (18, 19).

Previously we showed that PTX3 negatively regulates primary immunity to *L. major* by suppressing Th17 responses (11). Mechanistically, PTX3 suppresses the production of IL-6 and TGF-β by dendritic cells and expression of Th17 transcription factors such as RORγt, STAT3 and AhR in CD4^+^ T cells (11). Thus, in *PTX3^-/-^* mice, the absence of this regulation leads to increased production of IL-6 and TGF-β by infected DCs (11), favoring optimal differentiation of CD4^+^ T cells into Th17 cells leading to the enhanced production of IL-17A. Several cell types, including γδ T lymphocytes and group 3 innate lymphoid cells (ILC3s), are established sources of IL-17A and play critical roles in shaping immune responses during cutaneous leishmaniasis (24, 25). Although the present study did not directly assess the effect of PTX3 deficiency on IL-17A production from these populations, we observed IL-17A expression within the CD3^+^CD4^−^ compartment, most likely corresponding to CD8^+^ T cells. Notably, PTX3^−^/^−^ mice exhibited significantly higher IL-17A levels in this subset (data not shown), suggesting that PTX3 may exert broader regulatory control over IL-17A responses beyond CD4^+^ T cells and influence additional lymphoid lineages.

In healed mice, the development of a delayed-type hypersensitivity (DTH) response and resistance to reinfection depends on the presence of antigen-specific memory CD4^+^ T cells (22, 23), which can rapidly migrate to peripheral tissues at the site of antigen re-exposure (24). DTH is often used as a surrogate marker of vaccine-induced or infection-acquired immunity in cutaneous leishmaniasis (25). In line with this, we observed that healed *PTX3*^-/-^ mice exhibited significantly greater DTH response, a higher frequency of splenic effector memory CD4^+^ T cells (CD44^+^CD62L^-^), and reduced parasite burdens following secondary *L. major* challenge.

Development of memory T cells involves initial activation of naive T cells upon antigen encounter, followed by clonal expansion and acquisition of effector phenotype (26). Following antigen clearance and subsequent immune contraction, a small subset of effector T cells transition into memory phenotype and remain in circulation as either central and effector memory cells while some remain in tissues as tissue resident memory cells (27–30). Central memory T cells preferentially express CCR7 and CD127 and produce IL-2, whereas effector memory cells are poised for rapid IFN-γ production upon antigen restimulation (31–34) Since the quality and magnitude of memory T cell response is directly proportional to the magnitude of primary response, a primary infection must generate sufficient clonal T cell expansion to ensure the generation of optimal memory pool that mediates effective memory T cell response (26). We do not have any evidence suggesting that PTX3 influences initial CD4^+^ T cell clonal expansion following *L. major* infection, although we did find that healed PTX3 deficient mice contain significantly higher frequency of effector memory-like T cells in their spleens.

In our study, healed PTX3 deficient mice exhibited higher frequencies of effector memory CD4^+^ T cells in the spleen than their WT controls. This likely contributes to their enhanced recall responses manifested as increased DTH, proliferation and cytokine production upon secondary challenge. Flow cytometric analysis further supported this, showing that the P1 cluster (enriched for CD44^+^CD127^+^ cells) was expanded in PTX3 deficient mice. While the exact mechanism by which PTX3 modulates the memory CD4^+^ T cell pool remains to be defined, it is plausible that PTX3 inhibits key aspects of antigen presentation, such as costimulatory molecule expression or cytokine production, that are critical for memory T cell differentiation (28). Although the role of IL-17 in primary resistance to leishmaniasis is equivocal and may be dependent on parasite strain and species, numerous studies support a protective role for IL-17A in *L. infantum, L. major* and human post kala-azar dermal leishmaniasis (35–37). We previously showed that in PTX3 deficient animals, enhanced IL-17 response synergizes with IFN-γ to promote more efficient parasite clearance in macrophages during primary *L. major* infection (11). However, its role in secondary immunity has not been elucidated. Our current *in vivo* IL-17 neutralization studies demonstrated that akin to primary infection, PTX3 deficient mice lose their enhanced resistance to *L. major* challenge following IL-17A blockade, thereby confirming a critical role for IL-17A in mediating effective secondary immunity in cutaneous leishmaniasis.

In the current study, we observed that healed PTX3^−^/^−^ mice produced significantly higher levels of IL-10 than wild-type controls following secondary *L. major* challenge. This finding differs from our previous observations during primary infection, in which PTX3 deficiency was associated with markedly reduced IL-10 production (11). We propose that early attenuation of IL-10 responses in PTX3^−^/^−^ mice during primary infection may promote the development of a stronger effector T-cell pool and support more efficient parasite clearance (38, 39). Conversely, the elevated IL-10 detected in healed PTX3^−^/^−^ mice during secondary challenge may act as a counter-regulatory mechanism that tempers the accelerated Th1-driven recall response, thereby reducing the risk of immune-mediated tissue damage and limiting potential immunopathology (37).

The contribution of PTX3 to the pathogenesis of infectious diseases is context dependent and may vary with the nature of the pathogen and the affected tissue. PTX3 confers protection against several bacterial (40–43) and fungal (44) infections through modulation of host inflammatory and innate immune pathways. In contrast, our studies demonstrate that PTX3 promotes susceptibility to cutaneous leishmaniasis caused by *L. major*, largely by downregulating IL-17A production, which we found to enhance the development of a more effective CD4^+^ Th1 response (11). The role of PTX3 in visceral leishmaniasis caused by *L. donovani* or *L. infantum* has not yet been evaluated in our models. Because the immunopathological mechanisms underlying cutaneous and visceral disease are distinct, it is conceivable that PTX3 may play divergent, and potentially protective, functions in VL. Dedicated studies in experimental visceral leishmaniasis are therefore required to define how PTX3 regulates immunity across the different clinical forms of leishmaniasis.

We acknowledge several limitations of this work. First, the study was performed exclusively in a murine model of cutaneous leishmaniasis, and it remains uncertain whether these findings can be fully recapitulated in humans. Second, only female mice were used, which may introduce sex-related bias because sex hormones are known to influence the outcome of CL (45) and also regulate PTX3 expression, including modulation by estrogen and progesterone (46). Consequently, the observed immunological phenotypes may be specific to a particular biological sex and hormonal milieu. Finally, the present study did not define the mechanisms by which PTX3 shapes the function of antigen-specific memory effector CD4^+^ T cells. We did not employ T-cell receptor transgenic models in which CD4^+^ lymphocytes exclusively recognize a defined *Leishmania* antigen and therefore could not directly assess parasite-specific recall responses at the clonal level. Further studies using such reductionist systems, together with human validation, are required to delineate how PTX3 regulates protective memory immunity.

In summary, our findings clearly highlight the importance of PTX3 and IL-17A in regulating secondary immunity to cutaneous leishmaniasis. We identify PTX3 as a negative regulator of IL-17A-mediated secondary immunity to *L. major*. In its absence, CD4^+^ T cells expand into effector memory pool capable of mounting robust IL-17A responses leading to improved protection upon reinfection. These findings have important implications for vaccine development and suggest that targeting PTX3 or enhancing IL-17A responses could serve as a strategy to boost long-term protective immunity against cutaneous leishmaniasis.

## Data Availability

The original contributions presented in the study are included in the article/**Supplementary Material**. Further inquiries can be directed to the corresponding author.

## Supplementary material

Supplementary Figure 1 PTX3 deficiency does not affect TNF-α production during secondary *L. major* infection. Healed and age-matched naïve wild-type (WT) and PTX3^−^/^−^ mice were infected in the footpad (contralateral for healed mice) with 1×10^6^ stationary-phase *L. major* promastigotes Three weeks post-infection, mice were sacrificed, and the frequency of TNF-α^+^ -producing CD4^+^ T cells in the spleen **(A, B)** and dLNs **(A, C)** was assessed *ex vivo* by flow cytometry, gating on CD3^+^CD4^+^ cells. Additionally, spleen **(D)** and dLN **(E)** cells were restimulated *in vitro* with soluble *Leishmania* antigen (SLA; 50 μg/mL) for 72 hours, and TNF-α levels in the culture supernatants were measured by ELISA. Data are presented as means ± standard error and are representative of 2 independent experiments (n = 5 mice per group) with similar results. ns, Not significant.

Supplementary Figure 2 PTX3 deficiency does not affect IL-10 production during secondary *L. major* infection. Healed and age-matched naïve wild-type (WT) and PTX3^−^/^−^ mice were infected in the footpad (contralateral for healed mice) with 1×10^6^ stationary-phase *L. major* promastigotes Three weeks post-infection, mice were sacrificed and the frequency of IL-10^+^ -producing CD4^+^ T cells in the spleen **(A, B)** and dLNs **(A, C)** was analyzed directly *ex vivo* by flow cytometry by gating on CD3^+^CD4^+^ T cells. Additionally, spleen **(D)** and dLN **(E)** cells were restimulated *in vitro* with SLA (50 μg/mL) for 72 hours, and IL-10 levels in the culture supernatants were measured by ELISA. Data are presented as means ± standard error and are representative of 2 independent experiments (n = 5 mice per group) with similar results. *p < 0.05; ****p < 0.0001; ns, Not significant.

Supplementary Figure 3 PTX3 deficiency does not affect the frequency of CD4^+^CD25^+^FoxP3^+^ (regulatory T cells during secondary *L. major* infection. Healed and age-matched naïve wild-type (WT) and PTX3^−^/^−^ mice were infected in the footpad (contralateral for healed mice) with 1×10^6^ stationary-phase *L. major* promastigotes. Three weeks postinfection, mice were sacrificed and single cell suspensions were prepared from spleen **(A, B)** and dLN **(A, C)**. The frequency of FoxP3^+^CD25^+^ cells (regulatory T cells) was analyzed directly *ex vivo* by flow cytometry, gating on CD3^+^CD4^+^ T cells. Data are presented as means ± standard error and are representative of 2 independent experiments (n = 5 mice per group) with similar results. ns, Not significant.

Supplementary Figure 4 PTX3 deficiency enhances *in vivo* recall of antigen experienced Ki67^+^ IL-17^+^ CD4^+^ T cells during secondary *L. major* infection. WT and *PTX3*^-/-^ were infected in the footpad with 1x10^6^ stationary-phase *L. major* promastigotes and allowed to heal (> 12 weeks). Healed mice and age-matched naïve controls were challenged with *L. major* (contralateral footpad for healed mice), and sacrificed on Day 7 post-challenge. Draining lymph node cells were analyzed directly *ex vivo* by flow cytometry. Proliferating CD4^+^ T cells (Ki67^+^CD4^+^) were assessed **(A, B)**, and the frequency of proliferating, cytokine-producing CD4^+^ T cells was determined by co-expression of Ki67 and IFN-γ **(C, D)** or IL-17A **(E, F)**, gating first on CD3^+^CD4^+^ cells followed by analysis of Ki67^+^ subsets. Data are presented as means ± standard error and are representative of 2 independent experiments (n = 3 mice per group) with similar results. *, *p* < 0.05; ns, Not significant.

Supplementary Figure 5 Gating strategy for Memory CD4^+^ T cells. Splenic or draining lymph node cells were first gated on FSCA (x-axis) and SSCA (y-axis) to select lymphocytes. These lymphocytes were gated on FSCA (x-axis) and FSCH (y-axis) to select singlets. These singlets were then gated on CD3 (x-axis) and CD4 (y-axis) to select double positive CD3^+^ CD4^+^ T cells. These were then gated on CD62L (x-axis) and CD44 (y-axis) to resolve them into effector memory (CD44^+^CD62L^-^) CD4^+^ T cells.

Supplementary Figure 6 Gating strategy for cytokine producing CD4^+^ T cells and T regulatory cells. Splenic or draining lymph node cells were first gated on FSCA (x-axis) and SSCA (y-axis) to select lymphocytes. These lymphocytes were gated on FSCA (x-axis) and FSCH (y-axis) to select singlets. These singlets were then gated on CD3 (x-axis) and CD4 (y-axis) to select double positive CD3^+^ CD4^+^ T cells. These were then gated on IFN-γ^+^ CD4^+^ T cells, IL-10^+^ CD4^+^ T cells, TNF-α^+^ CD4^+^ T cells, IL-17^+^ CD4^+^ T cells, FOXP3^+^ CD25^+^ CD4 ^+^ T cells.

Supplementary Figure 7 Gating strategy for assessing *ex vivo* proliferation and cytokine producing CD4^+^ T cells in BrdU and Ki67 stained cells. Splenic or draining lymph node cells were first gated on FSCA (x-axis) and SSCA (y-axis) to select lymphocytes. These lymphocytes were gated on FSCA (x-axis) and FSCH (y-axis) to select singlets. These singlets were then gated on CD3 (x-axis) and CD4 (y-axis) to select double positive CD3^+^ CD4^+^ T cells. These were then gated on proliferating BrdU^+^ CD4^+^ T cells **(A)** or Ki67^+^ CD4^+^ T cells **(B)**. These proliferating CD4+ T cells were further gated on IFN-γ secreting cells (IFN-γ^+^ BrdU^+^ or IFN-γ^+^ Ki67^+^) and IL-17A secreting cells (IL-17A^+^ BrdU^+^ or IL-17A^+^ Ki67^+^).
