# Correction: Long pentraxin 3 (PTX3) regulates IL-17A-mediated secondary immunity to *Leishmania major* infection in mice

**DOI:** 10.3389/fimmu.2026.1817792

**Published:** 2026-03-18

**Authors:** Gaurav Gupta, Zhirong Mou, Ping Jia, Chukwunonso Onyilagha, Lianyu Shan, Abdelilah Soussi-Gounni, Jude E. Uzonna

**Affiliations:** 1 Department of Immunology, Max Rady College of Medicine, University of Manitoba, Winnipeg, MB, Canada; 2 Department of Medical Microbiology & Infectious Diseases, Max Rady College of Medicine, University of Manitoba, Winnipeg, MB, Canada; 3 Department of Pathology, Max Rady College of Medicine, University of Manitoba, Winnipeg, MB, Canada

**Keywords:** IL-17A, *Leishmania*, memory cells, PTX3, secondary immunity

The title of this article was erroneously given as: Long pentraxin 3 (PTX3) regulates IL-17A-mediated immunity to *Leishmania major* infection in mice.

The correct title of the article is: “Long pentraxin 3 (PTX3) regulates IL-17A-mediated secondary immunity to *Leishmania major* infection in mice.”

In the published article, there was a mistake as we have missed including a sentence in the Introduction section (changes in the 2nd and 3rd paragraph in the Introduction section) of the manuscript without which the sentence looks incomplete and inconclusive.”

A correction has been made to the section: **Introduction**, Paragraph Number 2 & 3:

“PTX3 is an acute phase inflammatory protein, comprising of a signal peptide, an N-terminal domain unrelated to any known protein, and a C-terminal pentraxin domain homologous to the short pentraxins, CRP and SAP (6, 7). Expressed by immune and non-immune cells (8), it has a dual role in infection. While protective against bacterial, viral, and fungal pathogens, PTX3 has also been implicated in disease susceptibility (9, 10).

We recently showed that PTX3 is induced during *L. major* infection and suppresses Th17 responses without affecting Th1 immunity (11). Thus, *PTX3*-deficient (*PTX3^-/-^*) mice show enhanced resistance to primary *L. major* infection compared to wild-type controls (11). Although this suppression hinders primary immunity, its role in regulating secondary immunity remains unclear. Furthermore, if this enhanced primary immunity does not translate to robust secondary immunity, targeting PTX3 may not be a viable therapeutic approach for individuals in endemic regions who face repeated exposures to *Leishmania*.”

The original version of this article has been updated.

