# Supplementary figures and images for: Long pentraxin 3 (PTX3) regulates IL-17A-mediated secondary immunity to Leishmania major infection in mice
Source: Front Immunol. 2026 Feb 10;17:1740323. doi: 10.3389/fimmu.2026.1740323 (PMC12929434; doi:10.3389/fimmu.2026.1740323)

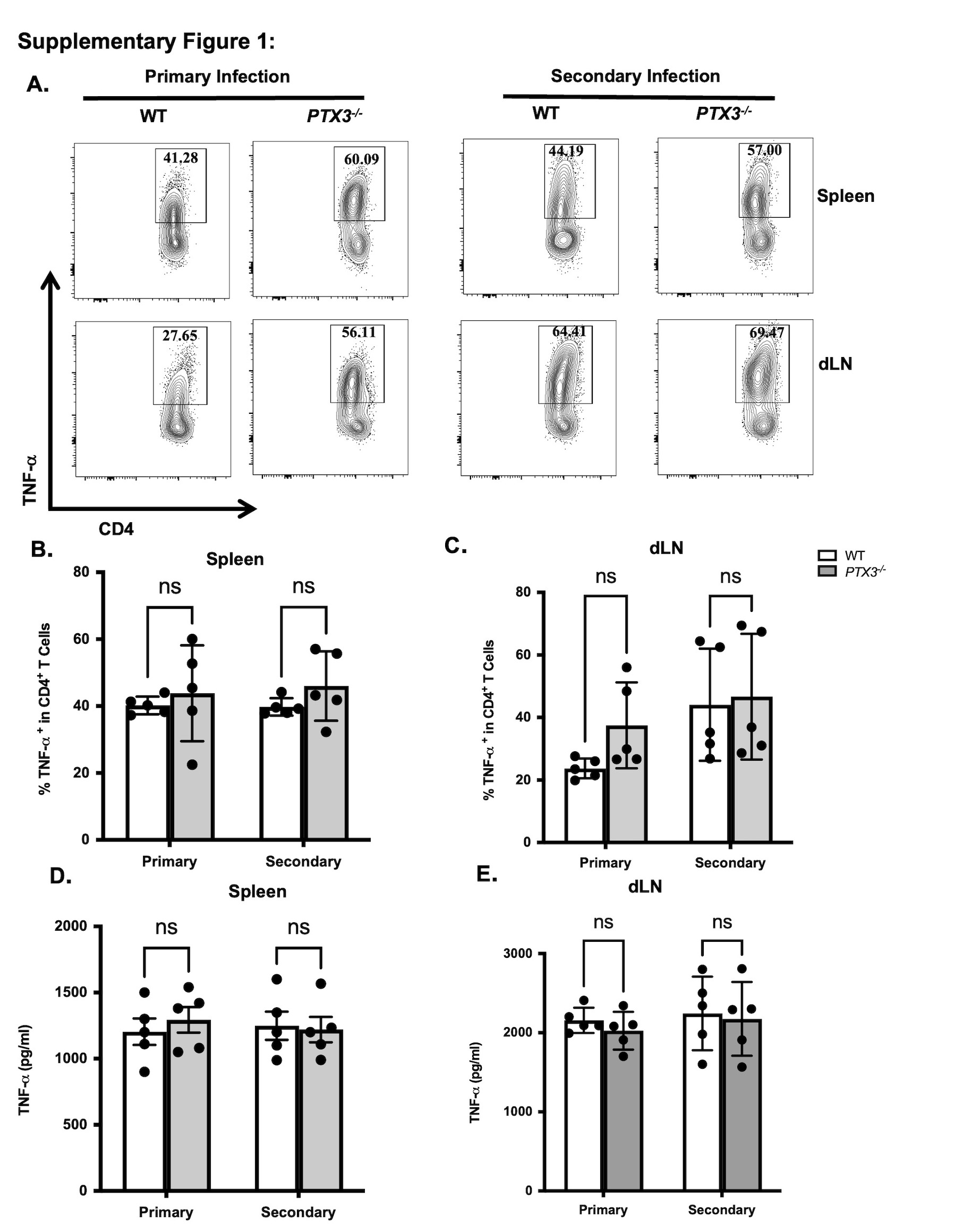

Supplement: Supplementary Figure 1 — PTX3 deficiency does not affect TNF-α production during secondary L. major infection. Healed and age-matched naïve wild-type (WT) and PTX3−/− mice were infected in the footpad (contralateral for healed mice) with 1×10^6^ stationary-phase L. major promastigotes Three weeks post-infection, mice were sacrificed, and the frequency of TNF-α+ -producing CD4+ T cells in the spleen (A, B) and dLNs (A, C) was assessed ex vivo by flow cytometry, gating on CD3+CD4+ cells. Additionally, spleen (D) and dLN (E) cells were restimulated in vitro with soluble Leishmania antigen (SLA; 50 μg/mL) for 72 hours, and TNF-α levels in the culture supernatants were measured by ELISA. Data are presented as means ± standard error and are representative of 2 independent experiments (n = 5 mice per group) with similar results. ns, Not significant. [file Image1.jpeg]

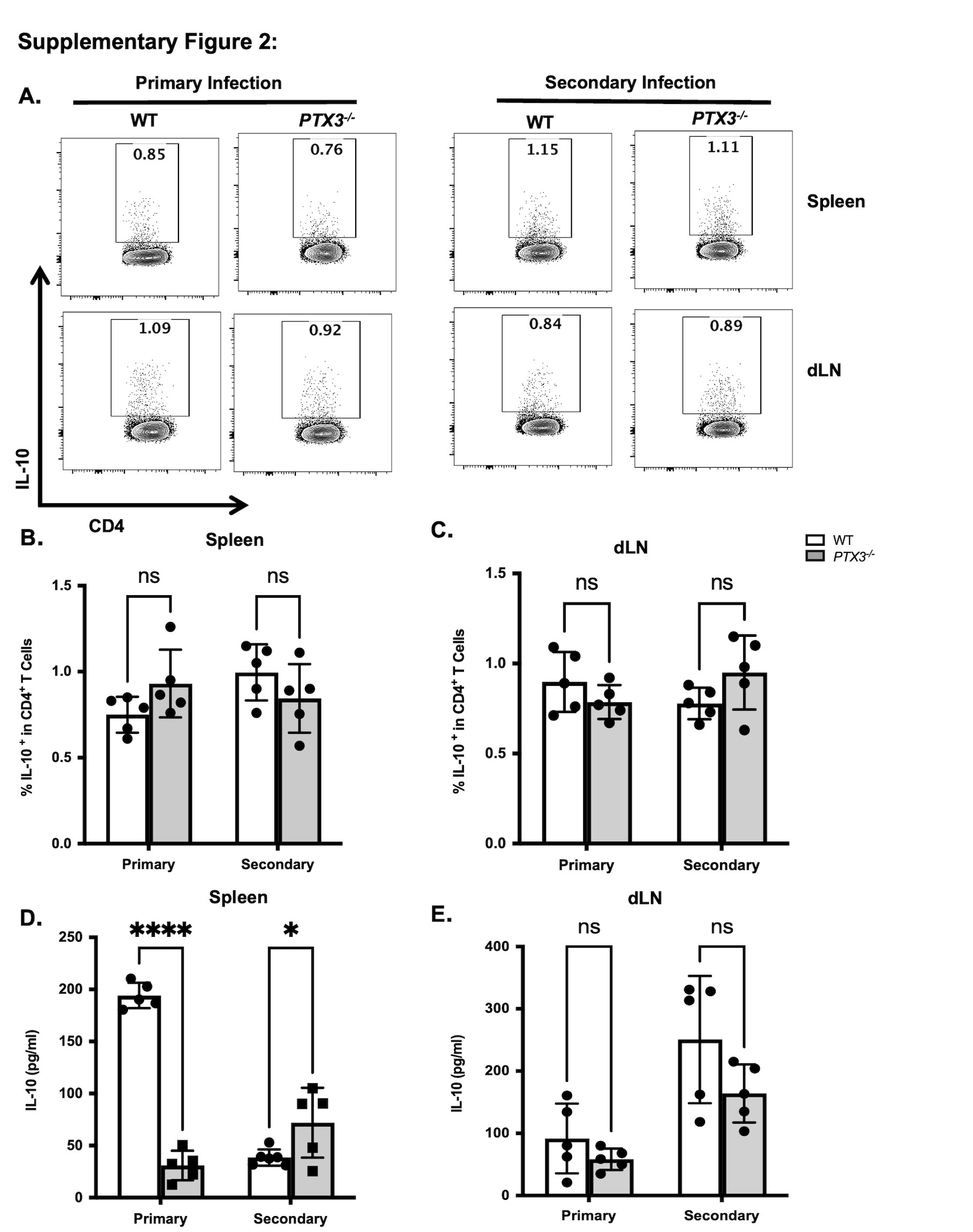

Supplement: Supplementary Figure 2 — PTX3 deficiency does not affect IL-10 production during secondary L. major infection. Healed and age-matched naïve wild-type (WT) and PTX3−/− mice were infected in the footpad (contralateral for healed mice) with 1×10^6^ stationary-phase L. major promastigotes Three weeks post-infection, mice were sacrificed and the frequency of IL-10+ -producing CD4+ T cells in the spleen (A, B) and dLNs (A, C) was analyzed directly ex vivo by flow cytometry by gating on CD3+CD4+ T cells. Additionally, spleen (D) and dLN (E) cells were restimulated in vitro with SLA (50 μg/mL) for 72 hours, and IL-10 levels in the culture supernatants were measured by ELISA. Data are presented as means ± standard error and are representative of 2 independent experiments (n = 5 mice per group) with similar results. *p < 0.05; ****p < 0.0001; ns, Not significant. [file Image2.jpeg]

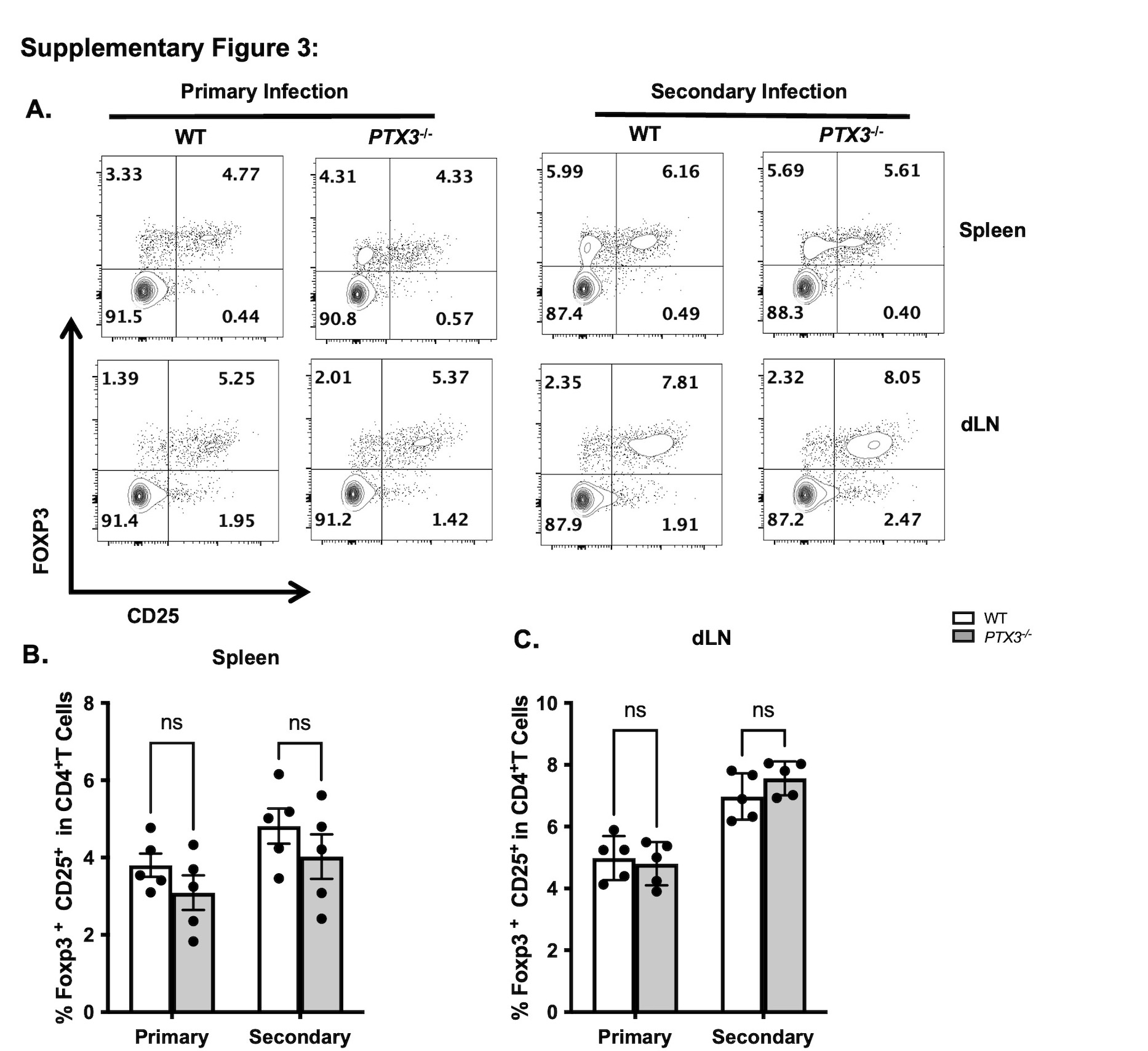

Supplement: Supplementary Figure 3 — PTX3 deficiency does not affect the frequency of CD4+CD25+FoxP3+ (regulatory T cells during secondary L. major infection. Healed and age-matched naïve wild-type (WT) and PTX3−/− mice were infected in the footpad (contralateral for healed mice) with 1×10^6^ stationary-phase L. major promastigotes. Three weeks postinfection, mice were sacrificed and single cell suspensions were prepared from spleen (A, B) and dLN (A, C). The frequency of FoxP3+CD25+ cells (regulatory T cells) was analyzed directly ex vivo by flow cytometry, gating on CD3+CD4+ T cells. Data are presented as means ± standard error and are representative of 2 independent experiments (n = 5 mice per group) with similar results. ns, Not significant. [file Image3.jpeg]

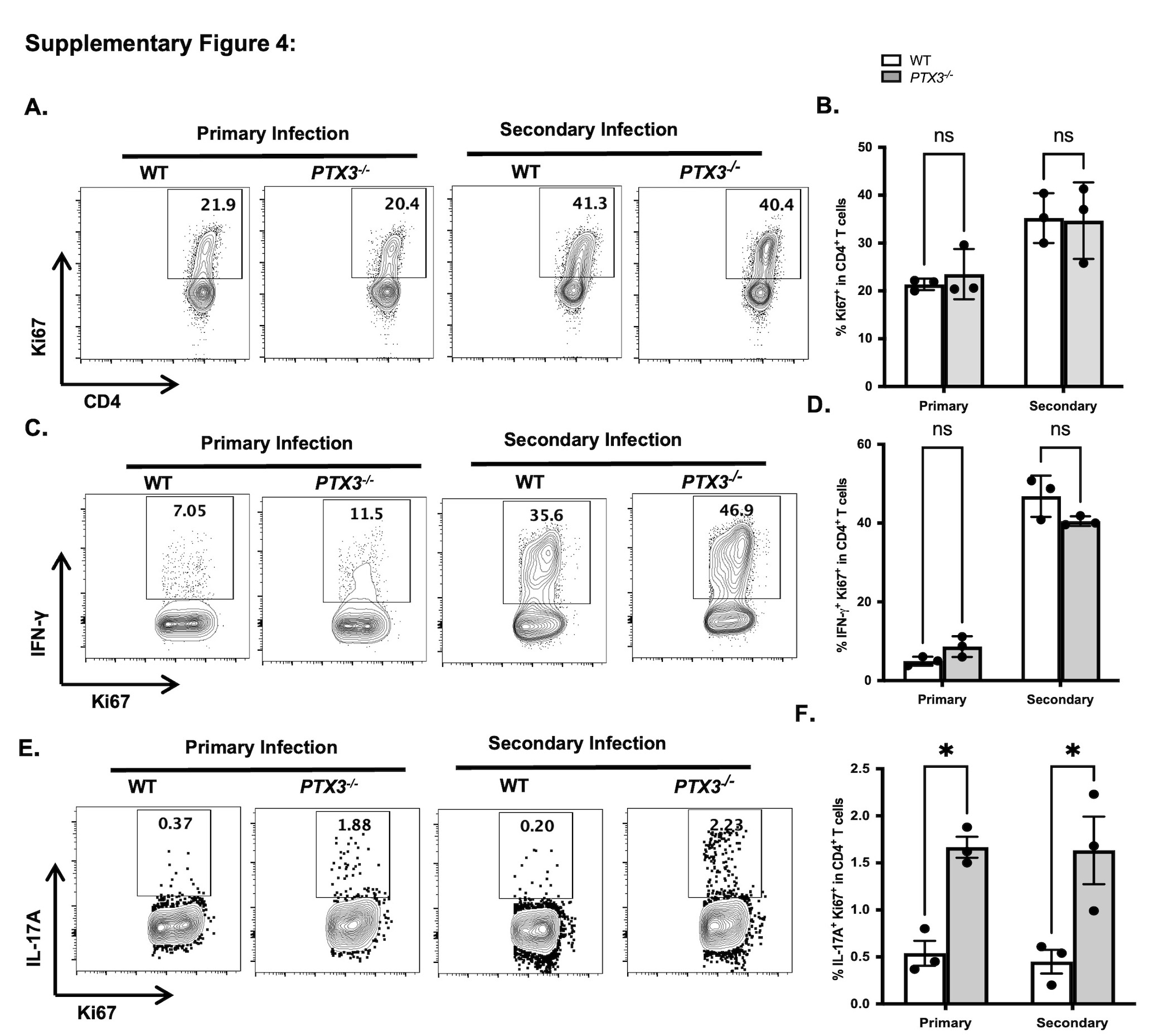

Supplement: Supplementary Figure 4 — PTX3 deficiency enhances in vivo recall of antigen experienced Ki67+ IL-17+ CD4+ T cells during secondary L. major infection. WT and PTX3-/- were infected in the footpad with 1x10^6^ stationary-phase L. major promastigotes and allowed to heal (> 12 weeks). Healed mice and age-matched naïve controls were challenged with L. major (contralateral footpad for healed mice), and sacrificed on Day 7 post-challenge. Draining lymph node cells were analyzed directly ex vivo by flow cytometry. Proliferating CD4+ T cells (Ki67+CD4+) were assessed (A, B), and the frequency of proliferating, cytokine-producing CD4+ T cells was determined by co-expression of Ki67 and IFN-γ (C, D) or IL-17A (E, F), gating first on CD3+CD4+ cells followed by analysis of Ki67+ subsets. Data are presented as means ± standard error and are representative of 2 independent experiments (n = 3 mice per group) with similar results. *, p < 0.05; ns, Not significant. [file Image4.jpeg]

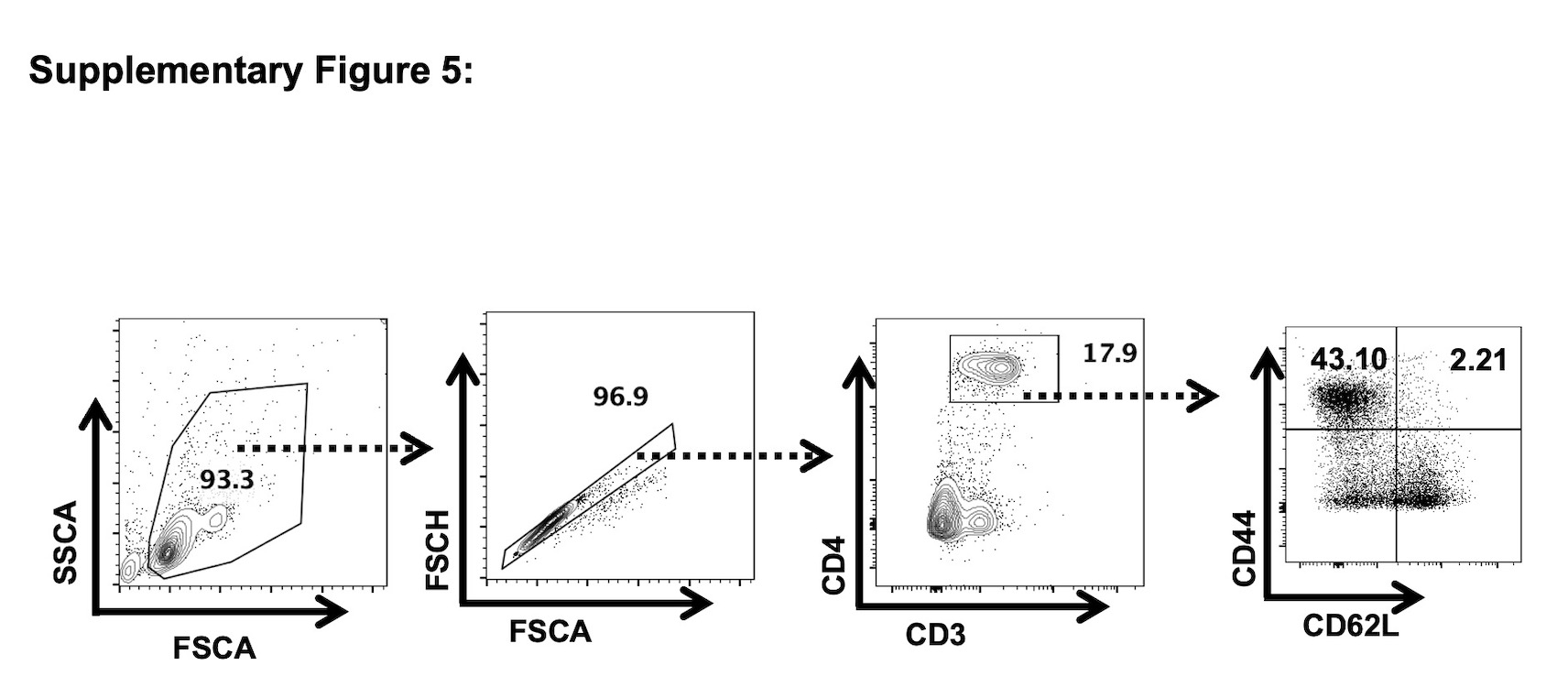

Supplement: Supplementary Figure 5 — Gating strategy for Memory CD4+ T cells. Splenic or draining lymph node cells were first gated on FSCA (x-axis) and SSCA (y-axis) to select lymphocytes. These lymphocytes were gated on FSCA (x-axis) and FSCH (y-axis) to select singlets. These singlets were then gated on CD3 (x-axis) and CD4 (y-axis) to select double positive CD3+ CD4+ T cells. These were then gated on CD62L (x-axis) and CD44 (y-axis) to resolve them into effector memory (CD44+CD62L-) CD4+ T cells. [file Image5.jpeg]

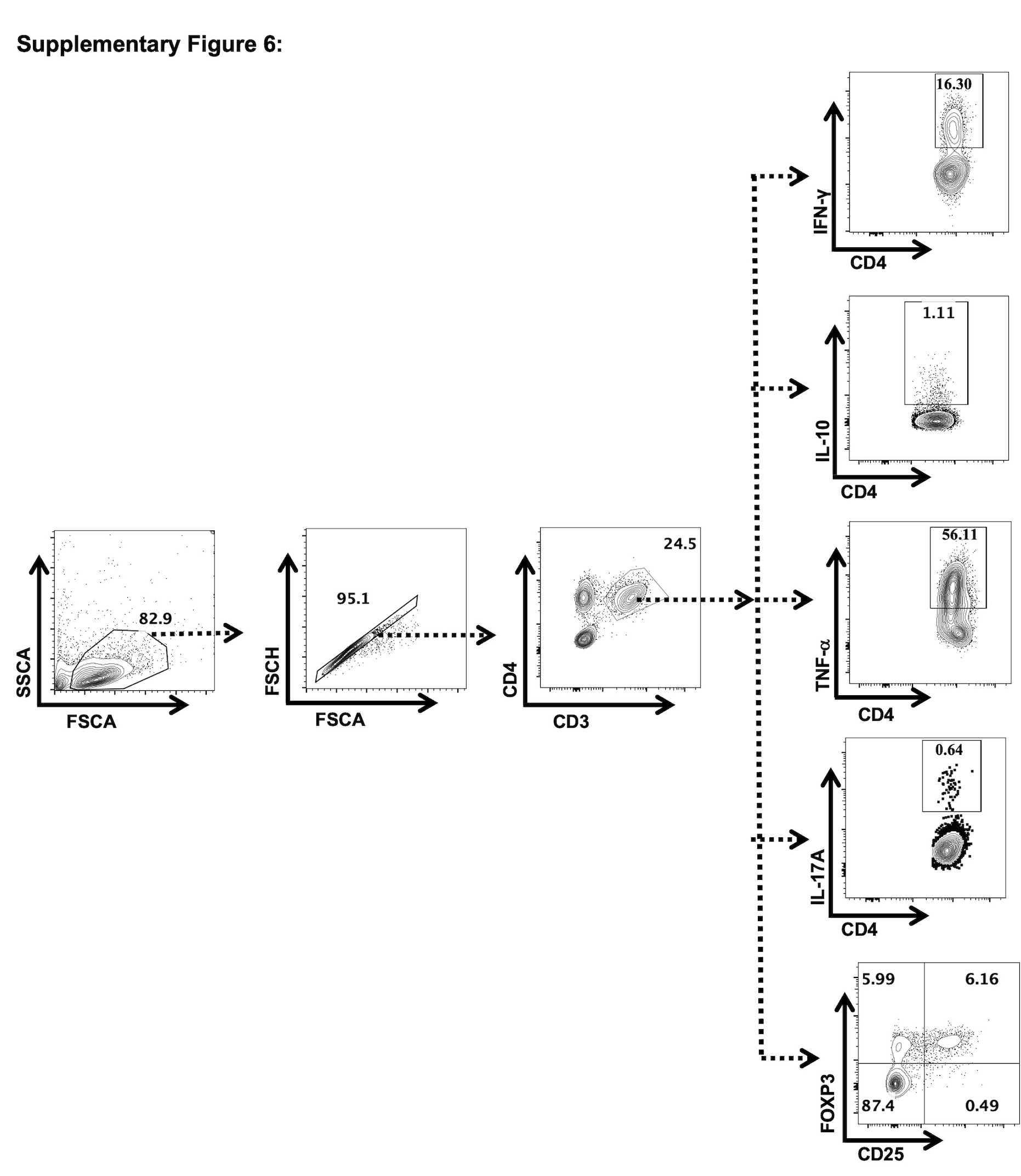

Supplement: Supplementary Figure 6 — Gating strategy for cytokine producing CD4+ T cells and T regulatory cells. Splenic or draining lymph node cells were first gated on FSCA (x-axis) and SSCA (y-axis) to select lymphocytes. These lymphocytes were gated on FSCA (x-axis) and FSCH (y-axis) to select singlets. These singlets were then gated on CD3 (x-axis) and CD4 (y-axis) to select double positive CD3+ CD4+ T cells. These were then gated on IFN-γ+ CD4+ T cells, IL-10+ CD4+ T cells, TNF-α+ CD4+ T cells, IL-17+ CD4+ T cells, FOXP3+ CD25+ CD4 + T cells. [file Image6.jpeg]

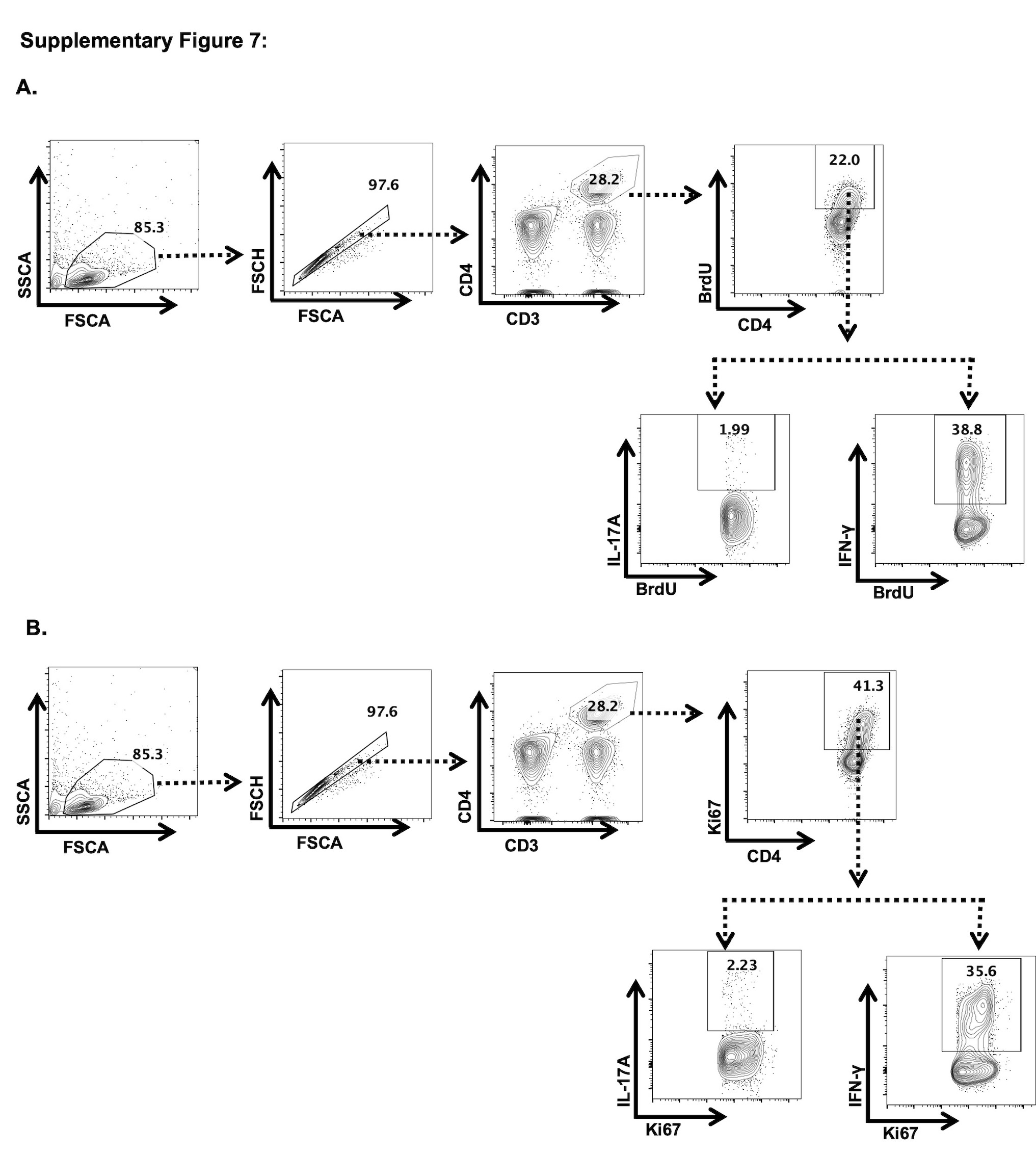

Supplement: Supplementary Figure 7 — Gating strategy for assessing ex vivo proliferation and cytokine producing CD4+ T cells in BrdU and Ki67 stained cells. Splenic or draining lymph node cells were first gated on FSCA (x-axis) and SSCA (y-axis) to select lymphocytes. These lymphocytes were gated on FSCA (x-axis) and FSCH (y-axis) to select singlets. These singlets were then gated on CD3 (x-axis) and CD4 (y-axis) to select double positive CD3+ CD4+ T cells. These were then gated on proliferating BrdU+ CD4+ T cells (A) or Ki67+ CD4+ T cells (B). These proliferating CD4+ T cells were further gated on IFN-γ secreting cells (IFN-γ+ BrdU+ or IFN-γ+ Ki67+) and IL-17A secreting cells (IL-17A+ BrdU+ or IL-17A+ Ki67+). [file Image7.jpeg]
